# Risk factors for herpes zoster: should people with asthma or COPD be vaccinated?

**DOI:** 10.1186/s12931-022-02305-1

**Published:** 2023-01-28

**Authors:** Ekaterina Safonova, Barbara P. Yawn, Tobias Welte, Chengbin Wang

**Affiliations:** 1 GSK Vaccines, Moscow, Russia; 2 University of Minnesota, Minneapolis, MN USA; 3 Hannover School of Medicine and German Center for Lung Research, Hannover, Germany; 4 GSK Vaccines, Rockville, MD USA; 5 Present Address: Novavax Inc., Gaithersburg, MD USA

**Keywords:** Asthma, COPD, Herpes zoster, Shingles, Vaccine, Obstructive lung diseases, Prevention

## Abstract

Without vaccination, an estimated 1 in 3 individuals will develop herpes zoster (HZ) in their lifetime. Increased risk of HZ is attributed to impaired cell-mediated immunity, as observed in age-related immunosenescence or in individuals immunocompromised due to disease or immunosuppressive treatments. Most vaccination guidelines recommend HZ vaccination in all adults ≥ 50 years of age, although Shingrix^®^ was recently approved by the U.S. Food and Drug Administration for use in individuals aged ≥ 18 years who are or will be at increased risk of HZ due to immunodeficiency or immunosuppression caused by known disease or therapy, followed by approval by the European Medicines Agency for use in immunocompromised individuals aged ≥ 18 years. Chronic respiratory diseases are also risk factors for HZ. A new meta-analysis reported 24% and 41% increased risks of HZ in those with asthma and chronic obstructive pulmonary disorder (COPD), respectively, compared with healthy controls. Asthma and COPD increase a person’s risk of HZ and associated complications at any age and may be further elevated in those receiving inhaled corticosteroids. Despite the increased risks, there is evidence that HZ vaccination uptake in those aged ≥ 50 years with COPD may be lower compared with the age-matched general population, potentially indicating a lack of awareness of HZ risk factors among clinicians and patients. The 2022 Global Initiative for Chronic Lung Disease report recognizes that Centers for Disease Control and Prevention recommended to vaccinate those aged ≥ 50 years against HZ, although health systems should consider the inclusion of all adults with asthma or COPD into their HZ vaccination programs. Further research into HZ vaccine efficacy/effectiveness and safety in younger populations is needed to inform vaccination guidelines.

**Graphical Abstract:**

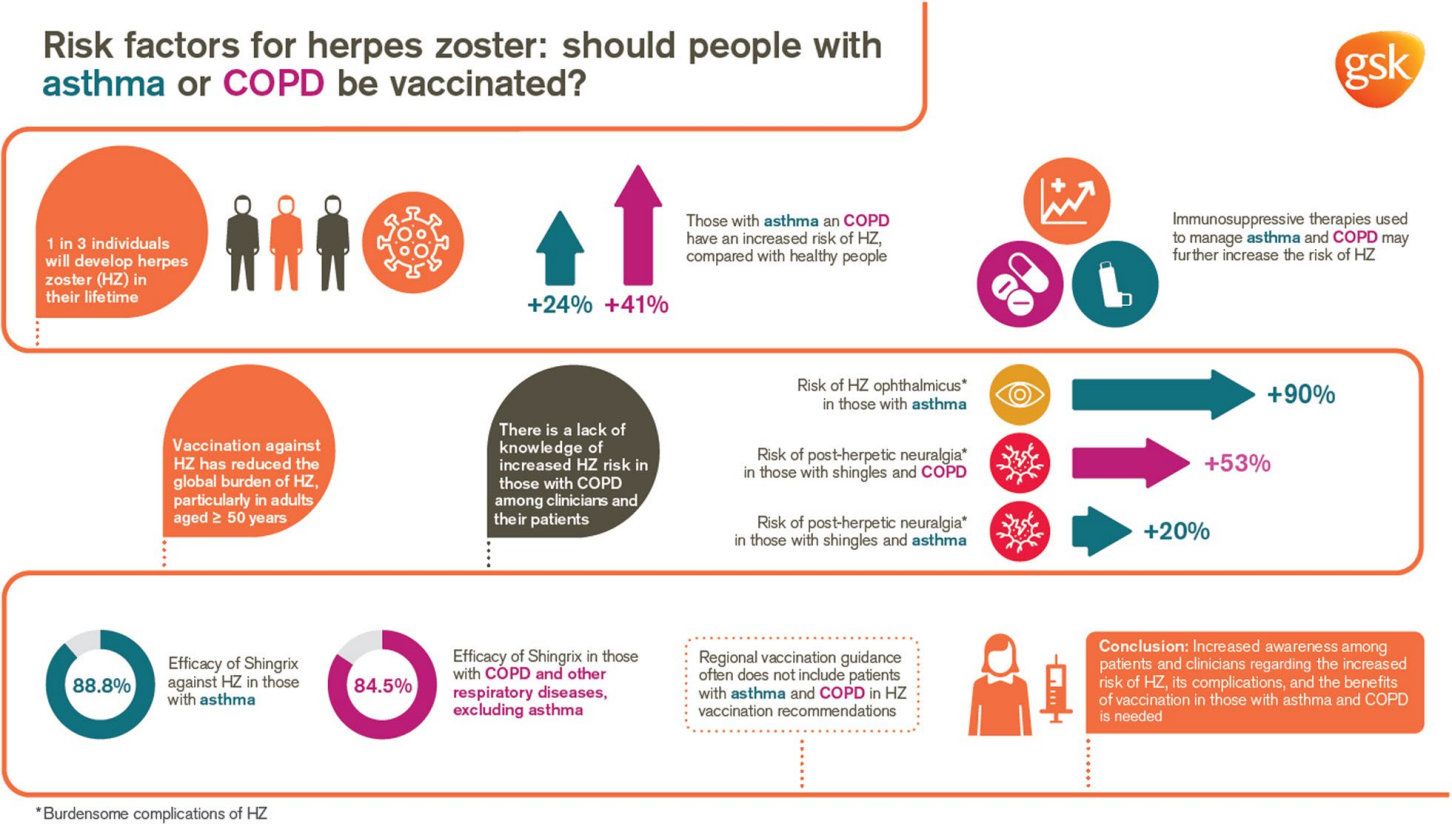

## Highlights

- Re-activation of the latent varicella zoster virus manifests as herpes zoster (HZ), which is associated with burdensome symptoms and can lead to complications
- Risk factors for HZ are often associated with a decline in cell-mediated immunity, as observed with ageing, immunocompromised conditions, immunosuppressive therapy and chronic conditions that compromise the immune system
- Several chronic conditions, including chronic respiratory conditions such as asthma and chronic obstructive pulmonary disorder (COPD), increase an individual’s risk of HZ and the associated complications
- HZ vaccination is effective at reducing HZ occurrence, with guidelines generally recommending their use in those ≥ 50 years of age and in at-risk adult populations
- There is some evidence that use of inhaled corticosteroids, which is often used to manage asthma or COPD, can independently increase a person’s risk of developing HZ
- The increased risk of HZ and its complications in those with asthma and COPD suggests that these populations may benefit from HZ vaccination, although cost–benefit analyses in those < 50 years of age are lacking

## Plain Language Summary

**What is the context?**

- After experiencing chickenpox, the varicella-zoster virus remains in the body and can be reactivated years later in a form called **herpes zoster**, more commonly known as **shingles**.
- Although shingles is more common in people aged ≥ 50 years, it is also more likely to occur in people with immune systems that do not work normally, which may include those with respiratory conditions such as **asthma** and **chronic obstructive pulmonary disorder (COPD)**.
- This disease can be prevented by **vaccination**. Therefore, it is important for doctors to know which patients are at increased risk of shingles and who could be considered for vaccination.

**What is new?**

- This review is the first to summarize the risk of shingles in people with asthma or COPD, drawing together evidence from across the world. It also evaluates the recommended use of different shingles vaccines in these patients, with a focus on two widely used vaccines: **Zostavax® (ZVL)** and **Shingrix® (RZV)**.
- Asthma or COPD can make people more likely to develop shingles and related medical complications, **even in younger people**.
- Most guidelines recommend vaccination against this disease for those aged 50 years and above, with some also recommending vaccination in people aged 18–49 years who may be at higher risk of shingles.
- There is limited information on the benefit of shingles vaccination in those aged ≤ 50 years with asthma or COPD, but their increased risk of developing shingles suggests they may also benefit from inclusion in vaccination programs.

**What is the impact?**

- The data presented in the review suggest that **people with asthma or COPD aged 18–49 years** could benefit from shingles vaccination.
- This group is not currently included in most vaccination guidelines, despite the evidence of increased risk of shingles and its complications. More information is needed on the **risks and benefits of vaccinating** this group to determine if it would be cost-effective.

## Background

Herpes zoster (HZ), or shingles, is a vaccine-preventable disease caused by the reactivation of latent varicella-zoster virus (VZV), which is present in > 95% of adults ≥ 40 years of age. The lifetime risk of shingles is > 30% in those not vaccinated against HZ [1–3]. Even with highly efficacious vaccines now available, HZ and its complications are a significant cause of morbidity, with an estimated 1 million cases per year in the United States [4].

Before vaccination programs, complications of HZ were reported in almost 1 in 4 patients with HZ [5]. The most common complication is post-herpetic neuralgia (PHN), which can significantly impact quality of life for months or years. It occurs in 5–30% of all HZ cases, with risk increasing with age; 80% of all PHN cases occur in those ≥ 50 years of age [6, 7]. HZ ophthalmicus (HZO) is the second most common complication of HZ, occurring in up to 10% of cases and may lead to severe outcomes such as blindness [8].

Many risk factors for HZ have been identified, including age, sex, family history of zoster, race, immunocompromised conditions (transplantation, human immunodeficiency virus [HIV], malignancies, and autoimmune diseases), and multiple chronic conditions [9, 10]. Increasing age is the leading risk factor for both HZ and PHN [11, 12] and is attributed to immunosenescence of cell-mediated immunity, specifically to the VZV. Ageing and many chronic conditions can compromise the immune system [13].

Having chronic conditions or comorbidity increases a person’s risk of developing HZ [9, 10]. On average, there is a 30% increased risk of acute HZ in those with at least one of the following conditions: asthma, chronic heart disease, chronic obstructive pulmonary disorder (COPD), depression and rheumatoid arthritis [14]. Both asthma and COPD have also been associated with an increased risk of PHN [11].

The reactivation of latent varicella-zoster virus is attributed to changes in cell-mediated immunity. Low levels of the T helper (Th) 1 cytokines interleukin (IL)-2 and tumor necrosis factor-α and high levels of the Th2 cytokines IL-10 and IL-4 have been observed in both the skin blisters and peripheral blood of patients with HZ [15, 16].

Asthma is associated with an imbalance in Th1 and Th2 immunity, with elevated Th2 and concomitant low Th1 immunity driving atopic and inflammatory chronic disorders. This low Th1 immunity could increase the risk of HZ infection. Innate immune pathways may also be impaired in asthma, with deficiencies in molecules of interferon-dependent and -independent antiviral signaling pathways reported, which may additionally increase risk of HZ [16, 17].

COPD results from interplay between cumulative exposure to noxious particles and gases, and host factors such as genetics or abnormal lung development [18]. The resulting chronic inflammation involves both the innate and adaptive immune system and is characterized by the presence of neutrophils, macrophages, and T and B lymphocytes [19].

Despite the well-documented increased risk of HZ and PHN in individuals with asthma or COPD, current guidelines for HZ vaccination programs often do not specifically include these respiratory diseases within their list of chronic conditions that should be considered for vaccination; therefore, individuals with respiratory diseases may not be covered in current recommendations for vaccination [20, 21].

Here we review the burden of HZ in adults with asthma or COPD, summarizing information that may support the development of future vaccination and disease guidelines. We use data and evidence from various countries to describe the risk of HZ in adults with asthma or COPD and discuss aspects to consider how these populations could benefit from prevention of HZ.

## Burden of HZ in chronic respiratory diseases: asthma and COPD

Asthma is a chronic disease characterized by inflammation and narrowing of the airways, which can lead to symptoms including cough, wheeze, shortness of breath and chest tightness. It was estimated to affect 262 million people worldwide in 2019 [22].

People with asthma have an increased susceptibility to both respiratory tract and non-respiratory tract viral and bacterial infections, and inflammatory diseases [23, 24]. These morbidities suggest a systemic nature of asthma and burden to the patient beyond airway inflammation [25]. Unidentified morbidities in patients with asthma may indirectly increase the risk of HZ infection [26]. For example, asthma is associated with a number of comorbid conditions such as COPD, diabetes, and depression [27–29], each of which are reported risk factors for HZ [9, 10].

In 2019, COPD was the third leading cause of death worldwide, with particular burden in low- and middle-income countries [30]. It is characterized by persistent respiratory symptoms and airflow limitations. The global prevalence of COPD may be increasing, with 10.7% reported in 1990, 11.7% in 2010, and 13.1% in 2019 [31].

COPD can increase healthcare resource utilization (HCRU) after HZ, compared with HZ cases without COPD. A study from Spain reported more medications, hospitalizations, and outpatient visits for HZ in those with COPD [32]. A recent US study also reported increased HCRU and costs associated with HZ in patients ≥ 50 years of age with COPD (n = 3415), compared with individuals with COPD alone (i.e., without HZ) (n = 35,360) [33]. This increase was in both all-cause and COPD-related use of medical services (inpatient, emergency department and outpatient) and costs in the year after HZ. Data from patient reports in the United States support this claim, with around 20% of patients with COPD reporting exacerbations or increased dyspnea associated with HZ [Barbara Yawn, unpublished data]. Studies have not evaluated whether HCRU and economic burden of HZ may increase in those with asthma.

Treatments are available to mitigate the symptoms of asthma and COPD. Inhaled corticosteroids (ICS) are used in both diseases to reduce inflammation of the lungs [22, 30]. For patients with asthma, the 2021 Global Initiative for Asthma (GINA) recommends that all adults and adolescents receive an ICS-containing controller treatment, as ICS is important for preventing severe exacerbations. Even for those with mild disease, an as-needed low-dose ICS-formoterol or, alternatively, the option of a regular low-dose ICS, plus as-needed short-acting beta-agonist, are recommended [34]. In contrast, for COPD, the Global Initiative for Chronic Obstructive Lung Disease (GOLD) only recommends the use of ICS in those with frequent exacerbations, and it is most effective in those with blood eosinophil count ≥ 300 cells per µL [18]. In this review, we will consider the potential risk factors for HZ, including age and corticosteroid therapy in asthma and COPD.

## Risk factors for HZ in patients with asthma

A systematic review and meta-analysis examined risk factors for HZ, excluding studies only evaluating immunosuppressive medication. The meta-analysis included 12 studies that assessed the risk of HZ in those with asthma, demonstrating a pooled relative risk (RR) of 1.24 (95% confidence interval [CI] 1.16–1.31; *P* < 0.0001). These data were adjusted for sex, age, and comorbidities [9].

### Age

Age is a key risk factor for HZ and is attributed to immunosenescence of cell-mediated immunity [11, 12]. The interaction of age and risk of HZ in those with asthma has been investigated in multiple studies.

In a population-based case–control study in Minnesota, USA (n = 371 HZ cases, n = 742 matched controls), there was an increased risk in HZ associated with asthma in all age categories (< 60 years, 60–69 years, ≥ 70 years), with similar strength of association across these age strata (*P* = 0.978) [35].

A matched cohort study in a Taiwanese population of 40,069 people with asthma, showed a significantly greater hazard ratio (HR; 1.48) for HZ in those with asthma compared with those without asthma in all age groups older than 21 years. The strength of association between asthma and HZ was similar across different age groups (21–40 years [adjusted HR: 1.71, 95% CI 1.22–2.40; *P* < 0.01], 41–60 years [adjusted HR: 1.42, 95% CI 1.23–1.65; *P* < 0.001], and ≥ 61 years [adjusted HR: 1.58, 95% CI 1.40–1.78; *P* < 0.001) [36].

A case–control study of UK primary care data from the Clinical Practice Research Database (CPRD) assessed 144,959 cases of HZ, of which 10,243 had a history of asthma. There was an increased risk of HZ in those with a variety of chronic conditions, including asthma (odds ratio [OR]: 1.21; 95% CI 1.17–1.25), when compared with matched controls. The increased risk of HZ in those with asthma was apparent in each age strata (< 50, 50–59, 60–69, ≥ 70 years) with a similar strength of association [37].

A retrospective, matched cohort study of German claims data, including 9 million people between 2008 and 2018, demonstrated an increased HZ risk in those with asthma in each age strata (18–49, 50–59 and ≥ 60 years) [14].

This was also recognized in a large study from Korea that analyzed data from 64,152 participants with HZ, of which 9728 had a history of asthma, aged between 20 years and ≥ 85 years. An increased risk of HZ was apparent in people with asthma across all age intervals [26].

Each of the studies described above suggest asthma increases the risk of HZ across all adult age groups, compared with age-matched populations without asthma. Two of these studies also assessed changes in HZ incidence across age groups in patients with asthma and found that, like in the general population, HZ increased with age [36, 37].

**Fig. 1:**
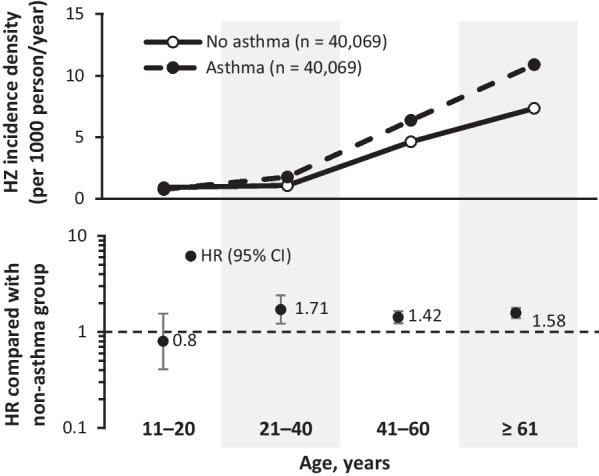
Incidence of HZ by asthma status. Compared with subjects without asthma, an increased incidence of HZ was observed in those with asthma across all adult age groups. Data from Peng et al. [36]. *CI* confidence interval, *HR* hazard ratio, *HZ* herpes zoster

Some studies have shown a higher RR of HZ in younger patients when compared with non-asthma age-matched controls [14, 26, 36]. This is likely driven by a much lower HZ incidence in the younger non-asthma population (Fig. 1).

### Corticosteroid use

Recent studies have suggested an association of corticosteroid use for the management of asthma with increased risk of HZ. This was observed in a retrospective analysis of claims data on the utilization of healthcare across Germany. This study reported a higher rate of HZ in those with asthma receiving ICS and oral corticosteroids, compared with an age-matched group not receiving ICS. This increased risk was observed across age groups (18–49, 50–59 and ≥ 60 years) [14]. In addition, the case–control study of UK primary care data reported an OR of 1.21 (95% CI 1.17–1.25) for HZ in those with asthma compared with matched controls, which was reduced to 1.11 (95% CI 1.06–1.16) after adjusting for ICS, oral corticosteroids, other immunosuppressive treatment [37].

In contrast, a US case–control study that included 87 cases of HZ in subjects with asthma did not find the use of ICS to be a risk factor for HZ, although statistical power may have been limited due to small sample size [35]. An analysis of data from the UK CPRD had similar conclusions. They assessed ICS use among 8900 incident cases of HZ in those with respiratory disorders, with a control cohort of patients with respiratory conditions matched based on age and calendar time of cohort entry. ICS use was not associated with risk of HZ (adjusted OR: 1.00; 95% CI 0.94–1.07), even at higher ICS doses (adjusted OR: 1.05; 95% CI 0.96–1.14). This cohort included 32.5% and 31.5% of patients with asthma in HZ cases and control cohorts, respectively. However, this study did not assess ICS adherence or stratify use of ICS by comorbid condition, so we cannot draw conclusions for patients with asthma from this analysis [38].

It is important to note that any association between corticosteroid use in asthma and HZ risk may be complicated by correlations between corticosteroid use and more severe asthma. In contrast to current recommendations [34], ICS was previously not recommended as a first-line therapy for mild asthma and was reserved for more severe asthma [39]. Therefore, corticosteroid use in the studies described above likely correlated with more severe asthma.

As far as we are aware, there are currently no data on whether use of biologics in asthma management is a risk factor for HZ.

## Risk factors for HZ in patients with COPD

Multiple studies have assessed the risk of HZ in patients with COPD. A recent meta-analysis, which included 12 studies assessing the RR of HZ in people with COPD, reported an RR of 1.41 (95% CI 1.28–1.55) [9]. Additionally, it has been observed that incidence of COPD exacerbations may be higher around the time of HZ episodes, with a spike in exacerbations observed in the month before HZ index date [33].

### Age

Multiple studies have demonstrated an increasing incidence rate of HZ in those with COPD across different ages. A retrospective analysis of a US claims database between 2013 and 2018 reported a higher incidence rate of HZ in those with COPD, compared with those without COPD, across all age strata investigated (40–49, 50–59, 60–69, 70–79, ≥ 80 years). The analysis included 39,816 patients with COPD and 2,575,007 patients without COPD [40].

**Fig. 2:**
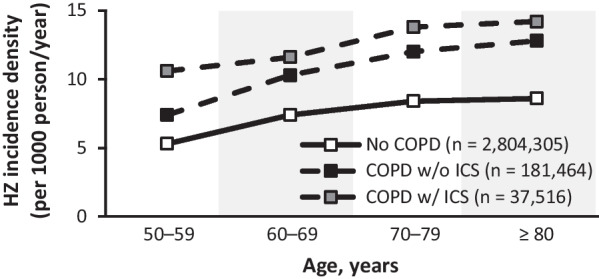
Incidence of HZ by COPD ± ICS status. Compared with those without COPD, increased incidence of HZ was observed in patients with COPD, and the use of ICS in those with COPD was associated with further increase in incidence, across all age groups assessed. Data from Muñoz-Quiles et al. [32]. *COPD* chronic obstructive pulmonary disorder, *HZ* herpes zoster, *ICS* inhaled corticosteroids, *w/* with, *w/o* without

Similarly, a population-based study that analyzed HZ incidence data from 161,317 patients with COPD in Spain between 2009 and 2014 also reported increasing HZ incidence across all ages in those with COPD (Fig. 2) [32].

The case–control study of UK primary care data included 144,959 cases of HZ, of which 6815 had a history of COPD. This study also reported an increased risk of HZ in those with COPD across age groups (< 50, 50–59, 60–69, ≥ 70 years). Additionally, they observed an increased adjusted OR of HZ across all age groups in those with COPD, compared with age-matched COPD controls from the general population; however, these did not individually reach statistical significance [37].

### Corticosteroid use

Studies have assessed the association of corticosteroid use and risk of HZ in patients with COPD. A population-based study from Taiwan investigated the risk of HZ in patients with COPD, and associated risk factors, in adults ≥ 50 years of age. They reported an adjusted HR of 1.67 (95% CI 1.43–1.96) for HZ in those with COPD who were not taking corticosteroid medication, compared with a matched control population (with no COPD and not taking steroids). When compared with the same control population, the risk was greater in those who were taking ICS (adjusted HR: 2.09; 95% CI 1.38–3.16) or oral (adjusted HR: 3.00; 95% CI 2.40–3.75) [41].

This finding was also supported by the analysis of UK primary care data, although they adjusted for all therapy and not corticosteroids alone. This study reported an adjusted HR of 1.32 (95% CI 1.27–1.37) for HZ in patients with COPD that, when additionally adjusted for oral and inhaled corticosteroids as well as other immunosuppressive therapy, reduced to 1.22 (95% CI 1.17–1.28), suggesting that the treatment of COPD can contribute to risk of HZ [37].

Additionally, a study in Spain assessed RR for HZ in adults ≥ 50 years old with COPD, who were either receiving or not receiving ICS, compared with a control population with no COPD. For those not receiving ICS, the adjusted RR was 1.45 (95% CI 1.41–1.5). For those receiving ICS, the adjusted RR rose to 1.61 (95% CI 1.52–1.71) (Fig. 2) [32].

Most recently, an analysis of administrative claims data from Germany between 2008 and 2018 showed an increased risk of HZ in those with COPD who had been prescribed at least one systemic corticosteroid in the year under study, compared with those who received no corticosteroids. The increased risk was observed in the 50–59 and ≥ 60 age groups but not the 18–49 age group, although this may have been influenced by small sample sizes in this age category [14].

The immunosuppressive action of inhaled and oral corticosteroids may increase susceptibility to infections through their effect on cellular immunity. This may be clearer in the case of systemic corticosteroids, with the effect of ICS on systemic immunity less clear.

Additionally, it is important to consider disease severity as a possible confounder when assessing steroid use and HZ risk in COPD, as these therapies are not recommended as a first-line treatment in mild or moderate COPD without exacerbations. More severe disease may also be contributing to an increased susceptibility to HZ in those receiving ICS for COPD, compared with those not receiving ICS who may have less severe COPD disease [32, 41].

## Risk and burden of HZ complications in those with asthma and COPD

Asthma or COPD may increase a person’s risk of developing PHN following HZ. A 2016 study of UK primary care data from the CPRD reported, among 119,413 patients with HZ, a 21% increased risk of PHN in those with asthma (95% CI 1.06–1.37) and 53% increased risk in those with COPD (95% CI 1.35–1.72) when adjusted for immunosuppressive therapy [11].

For HZO, a study of 137 patients at Kaiser Permanente Hawaii suggested that the presence of asthma may increase a person’s risk of developing HZO, reporting an OR of 1.9 (95% CI 1.1–3.2; *P* = 0.02) compared with those who did not have asthma [42]. Data are not available on the risk of HZO in those with COPD.

## The global use of zoster vaccines

### Approval of zoster vaccines

Zostavax^®^ (ZVL), a live attenuated vaccine, was the first vaccine to be licensed for use against HZ by the FDA in 2006 and was recommended for those ≥ 60 years of age. Since then, ZVL has been licensed in almost 60 countries [43]. It was later approved by both the FDA in 2011 and the European Medicines Agency (EMA) in 2012 for use in adults ≥ 50 years of age for the prevention of HZ and PHN [44, 45]. Contraindications for the vaccine include pregnancy, primary or acquired immunodeficiency states, and concurrent immunosuppressive therapy [44]. The latter does not include individuals who are receiving topical, inhaled, or low-dose systemic corticosteroids [46].

An adjuvanted recombinant zoster vaccine (RZV; Shingrix^®^) is increasingly being recommended in national vaccine guidelines and is currently licensed in over 37 countries [43]. The FDA approved the use of RZV in 2017 for use in adults ≥ 50 years of age. This was updated in July 2021 to include adults aged 18 years and older who are or will be at increased risk of HZ due to immunodeficiency or immunosuppression caused by known disease or therapy [47]. RZV was approved by the EMA in 2018 for use in adults ≥ 50 years of age and this indication was updated in September 2020 to include adults ≥ 18 years of age who are at increased risk of HZ [48].

Two other live attenuated vaccines have been approved for use. BIKEN^®^, which is licensed in Japan, contains a higher vOka titre than in ZVL and is indicated for the prevention of HZ in individuals ≥ 50 years of age. Sky Zoster^®^, a vaccine developed and licensed in Korea, has demonstrated non-inferiority to ZVL in healthy adults ≥ 50 years of age [43, 49, 50]. As clinical trial and real-world data are limited for these two vaccines, the rest of this review will focus on ZVL and RZV.

### Clinical trial data for zoster vaccines

Two clinical trials evaluated the efficacy of ZVL. The Shingles Prevention Study, which included over 38,000 subjects ≥ 60 years of age from the US, demonstrated an efficacy against HZ of 51.3% (95% CI 44.2–57.6). Vaccine efficacy was 37.6% in those aged ≥ 70 years and 63.9% in those aged 60–69 years. Additionally, the Shingles Prevention Study demonstrated a 66.5% (95% CI 47.5–79.2) reduction in the incidence of PHN in all vaccinated subjects, which was similar across the two age categories [51]. A separate study—ZEST (Zoster Efficacy and Safety Trial)—of 22,439 subjects aged 50–59 years from North America and Europe reported a ZVL vaccine efficacy of 69.8% (95% CI 54.1–80.6). Data for PHN were not available for this study [52].

Other studies have evaluated the real-world effectiveness of ZVL. A retrospective study of patients enrolled in the Kaiser Permanente Southern California health plan investigated HZ risk in 75,761 members vaccinated with ZVL and age-matched unvaccinated members. They reported a reduced risk of HZ in those vaccinated (HR = 0.45; 95% CI 0.42–0.48) [53]. A cohort study of health plan members vaccinated at Kaiser Permanente Northern California assessed ZVL effectiveness with an 8-year follow-up; 392,677 received the vaccine, with 9625 followed for the full 8 years. Vaccine effectiveness (VE) was 49.1% (95% CI 47.5–50.6), decreasing from 67.5% in year 1 to 31.8% in year 8 [54]. HZ rates were compared in ZVL vaccinated and unvaccinated populations in a study of US Medicare programs of 766,330 subjects ≥ 65 years of age. These data were similar to those observed in the ZVL clinical trials, with a real-world VE of 48% (95% CI 39–56) reported for adults ≥ 65 years of age [55]. In Australia, ZVL has been part of a national immunization program for those aged 70–79 since November 2016. A study assessed VE in the first 2 years of the program’s implementation, where 26,404 people were vaccinated across the 2 years. In the Year 1 group, which had an average time since vaccination of 8 months, the VE for ZVL was 63.5% (95% CI 47.5–74.6), which fell to 48.2% (95% CI 30.0–61.7) in the Year 2 group, which had an average time since vaccination of 18 months [56].

Two pivotal clinical trials investigated the efficacy and safety of RZV in people either ≥ 50 (ZOE-50) or ≥ 70 years of age (ZOE-70) [57, 58]. The ZOE-50 trial assessed efficacy by the age groups 50–59, 60–69, ≥ 70 years; vaccine efficacy against HZ in these age groups was 96.6% (95% CI 89.6–99.3), 97.4% (95% CI 90.1–99.7) and 97.9% (95% CI 87.9–100.0), respectively. Vaccine efficacy against PHN was also assessed. Pooled data from the ZOE-50 and ZOE-70 studies demonstrated a vaccine efficacy against PHN of 91.2% (95% CI 75.9–97.7) in those ≥ 50 years old and 88.8% (95% CI 68.7–97.1) in those ≥ 70 years old [58].

Real-world data are emerging on the VE of RZV. A retrospective US claims-based cohort study assessed HZ incidence in 173,745 adults who received two doses of RZV, comparing this to rates in almost 4.6 million unvaccinated controls. A VE of 86.8% (95% CI 86.4–88.7) was reported in individuals 50–79 years old and 80.2% (95% CI 75.1–84.3) in individuals ≥ 80 years old [59]. In a real-world retrospective analysis of immunocompetent individuals ≥ 50 years of age enrolled in Kaiser Permanente Hawaii health plan (11,864 individuals received two doses of RZV), RZV was 83.5% (95% CI 74.9–89.2) effective against HZ and 93.3% (95% CI 48.7–99.1) effective against HZO [60]. RZV was also evaluated in a 2-year post-marketing cohort study of US Medicare beneficiaries > 65 years of age (1,006,446 individuals received two doses of RZV). This real-world study found a VE of 70.1% (95% CI 68.6–71.5) for those receiving two doses and this was not significantly different in those > 80 years of age. In this study, VE against PHN in those receiving two doses was 76.0% (95% CI 68.4–81.8) [61]. Furthermore, two studies assessed RVZ in patients with inflammatory bowel disease (IBD) [62, 63]. There was a lower HZ infection risk in those with IBD ≥ 50 years of age who were vaccinated with RVZ (n = 1670) compared with those with IBD who were unvaccinated (n = 112,200) (OR = 0.36; 95% CI 0.23–0.56) [63].

Overall, these real-world studies report lower VE point estimates than the efficacy data reported in the pivotal ZOE studies; these variations are likely due to differences in case definition (laboratory confirmation was needed in clinical trials, leading to higher specificity and point estimate), study populations (clinical trials excluded those with immunosuppressive or immunodeficient disease, and those receiving immunosuppressive therapy, who are expected to have lower immune response), and age (≥ 50 years of age in the clinical trials) [57–61].

### ZVL and RZV efficacy, effectiveness, and real-world utilization in patients with respiratory diseases

An observational study investigated reasons for ZVL vaccine failure in adults in the UK ≥ 70 years of age [64]. They specifically looked at previously identified risk factors for HZ: age, sex, ethnicity, socio-economic status, asthma, diabetes, COPD, smoking, body mass index, immunosuppression, and history of HZ. In this observational cohort, overall VE was 66.8% (95% CI 62.2–71.0). The analysis identified a reduced VE in those with type 2 diabetes (−22.0% [95% CI −39.6, −4.5]) compared with those without diabetes, or with previous HZ infection (−22.5% [95% CI −44.9, −0.1]), compared with those with no history of HZ. Importantly, these two populations represent 28.5% of the entire population in which the vaccine is recommended. Neither asthma nor COPD influenced VE [64].

For RZV, a pooled exploratory post hoc analysis of the pivotal ZOE-50 and ZOE-70 clinical trials assessed the effect of medical conditions at baseline on the efficacy and safety of the vaccine. This analysis concluded that efficacy remained high in all groups with selected medical conditions, even in those with chronic conditions that increase the risk of HZ in unvaccinated populations, ranging between 84.5% and 97.0%. Efficacy was 88.8% (95% CI 63.6–97.8) in those with asthma and 84.5% (95% CI 46.4–97.1) in those with respiratory disorders excluding asthma. No safety concerns were identified in participants with any type of the selected medical conditions. Importantly, the study was not adjusted for multiplicity and its significance level was not controlled, furthermore the ZOE-50/-70 studies were not statistically powered to assess efficacy and safety by type of medical condition at baseline [65].

RZV is a two-dose vaccine series that gives a greater and more durable cell-mediated immunity response than a single dose [66]. Two-dose completion was evaluated in a study of 31,120 Kaiser Permanente Southern California members aged ≥ 50 years who had received a first RZV dose. Two-dose series completion was significantly lower in some groups at higher risk of HZ, such as older age groups and those with chronic conditions. COPD at baseline, along with several other chronic conditions, was associated with significantly lower series completion (*P* = 0.011). Series completion in those with baseline asthma was not investigated, and one-dose rates were not assessed [67].

The reduced uptake of a second RVZ dose is concerning and highlights a possible lack of awareness of HZ risk among both patients and healthcare professionals (HCPs). A US study of HCPs (n = 1020) suggests pulmonologists were the least likely of the HCPs studied to know that HZ vaccines are recommended for individuals ≥ 50 years of age, and were less likely to recommend a HZ vaccine compared with influenza or pneumococcal vaccines [68]. A separate study revealed only 25% of patients with COPD were aware that COPD increased the risk of developing HZ, with over 30% reporting the lack of HCP recommendation and lack of information as the reasons they had not received the HZ vaccine [69].

### Recommendation of RZV vaccines for asthma and COPD populations

The EMA and FDA recently expanded the RZV indication to include subjects ≥ 18 years of age who are at increased risk of HZ; the FDA specifically referenced those with immunosuppression or immunodeficiency caused by known disease or therapy [47, 70]. Similarly, the US Centers for Disease Control and Prevention (CDC) Advisory Committee of Immunization Practices (ACIP) recommends RVZ in immunodeficient or immunosuppressed adults ≥ 19 years of age, regardless of previous history of HZ infection or previous receipt of zoster vaccine [71]. There are other groups of adults aged 18–49 years at higher risk of HZ; however, the EMA Shingrix product information highlights the limited data in these populations and in individuals with a history of HZ [70].

Table 1 outlines country-specific guidelines on the use of RZV. Guidelines for the USA [72], Germany [73] and Italy [74] recommend the use of RZV in adults ≥ 50 years of age, including in those with chronic conditions. RZV is to be incorporated into the 2022 Swiss vaccination plan, with recommendations outlined in a bulletin published in 2021. Their recommendations are divided into three categories: immunocompetent adults ≥ 65 years of age, adults ≥ 50 years of age with current or future (cellular) immunodeficiency associated with an increased risk for HZ, and adults ≥ 18 years of age with a current severe immunodeficiency or an immunosuppressive treatment in the foreseeable future. Importantly, the group of adults ≥ 50 years of age includes those with asthma or COPD [75]. The German recommendations also mention COPD and asthma, which are listed among the risk groups in the ≥ 50 age category recommendation for RZV [76]. The Comité sur l'immunisation du Québec (CIQ) published updated recommendations on RZV for those 18–49 years of age and adults with chronic disease, to complement the recommendations already in place for all those ≥ 50 years of age. They recommend RZV in immunosuppressed people ≥ 18 years of age, including chronically ill people who meet the immunosuppression criteria. These criteria include those receiving immunosuppressive medications, such as corticosteroids. Importantly, the CIQ also suggests the vaccine could be considered in those ≥ 18 years of age with chronic diseases who are at higher risk of HZ, who may not be considered immunocompromised. Within this consideration they mention COPD and asthma as chronic diseases known to increase the risk of HZ [77].

**Table 1:** Overview of country and regional guidance for RZV vaccination, highlighting specific recommendations for adults with asthma and COPD

Regulator (country)	RZV vaccination recommendations	Refs.
CDC–ACIP (US)	**General population** Aged ≥ 50 years (including special populations e.g., chronic renal failure, diabetes mellitus, rheumatoid arthritis, and chronic pulmonary disease) **Immunocompromised adults** Aged ≥ 19 years for adults who are or will be immunodeficient or immunosuppressed because of disease or therapy	[71, 72]
CIQ (Canada –Quebec)	**General population** Aged ≥ 50 years and more particularly aged ≥ 65 years **Special population** • Aged ≥ 50 years with chronic diseases who are at high risk of shingles should be prioritized for vaccination • Aged ≥ 18 years for immunosuppressed adults and can be considered for adults with chronic diseases who are at high risk of shingles, without necessarily being immunocompromised or immunosuppressed (e.g., RA, SLE, chronic inflammatory bowel disease, COPD or bronchial asthma, chronic kidney disease and diabetes)	[77]
STIKO (Germany)	**General population** Aged ≥ 60 years **Special populations** Aged ≥ 50 years with increased health risk due to an underlying disease (e.g., congenital or acquired immunodeficiency or immunosuppression, HIV infection, RA, SLE, inflammatory bowel disease, COPD or bronchial asthma, chronic renal failure, or diabetes mellitus)	[73]
Ministero della Salut (Italy)	**General population** Aged ≥ 65 years, actively offered **Special populations** Aged ≥ 50 years, actively offered to those with diabetes mellitus, cardiovascular pathology, COPD, and subjects intended for immunosuppressive therapy	[74]
OFSP (Switzerland)	**General population** Aged ≥ 65 years who are immunocompetent **Special populations** • Aged ≥ 18 years with a current severe immunodeficiency or will receive an immunosuppressive treatment in the foreseeable future • Aged ≥ 50 years with a current or future (cellular) immunodeficiency associated with an increased risk for HZ (e.g., undergoing active cancer treatment; HIV; end-stage renal disease or on dialysis; receiving biologics, AZA, MTX or maintenance low-dose corticosteroid treatment; or with other underlying conditions affecting immunity including RA, severe asthma/COPD, inadequately controlled diabetes mellitus type 1 and other autoimmune diseases)	[75]

Guidelines for HZ vaccination with RZV vary between countries

*ACIP* Advisory Committee on Immunization Practices, *AZA*  azathioprine, *CDC*  US Centers for Disease Control and Prevention, *CIQ*  Comité sur l'immunisation du Québec, *COPD*  chronic obstructive pulmonary disorder, *HIV*  human immunodeficiency virus, *HZ*  herpes zoster, *MTX*  methotrexate, *OFSP* Office Fédéral de la Santé Publique (Federal Office of Public Health), *RA*  rheumatoid arthritis, *RZV*  recombinant zoster vaccine, *SLE*  systemic lupus erythematosus, *STIKO*  Standing Committee on Vaccination

Additionally, the GINA and GOLD reports discuss vaccination recommendations in those with asthma and chronic lung disease, respectively. Zoster vaccines are not mentioned in the 2021 GINA report [34]. The 2022 GOLD report added the recommendation of a zoster vaccine in those with COPD aged ≥ 50 years, citing the CDC recommendations [18].

## What does this mean?

Meta-analyses have robustly demonstrated an increased risk of HZ in those with asthma or COPD [9, 10], compared with those without asthma or COPD. Data also show that ICS use may be a separate risk factor for HZ in patients with these diseases. As for the general population, increasing age is also a risk factor in those with asthma or COPD [35, 40].

Recommendations for HZ vaccination align with the data and include those ≥ 50 years of age, which, possibly incidentally, encompassed the highest risk patient populations among those with asthma and COPD. However, it is worrying that vaccination against HZ might be low in those with respiratory diseases due to a lack of clinician and patient awareness of HZ risk factors [69], although this may improve following the recent inclusion of HZ vaccination in the GOLD 2022 recommendations for COPD [18].

Vaccination against HZ might be beneficial in those with asthma or COPD across a wide range of age groups, from young adults to elderly persons. Increased risk of HZ and it is complications in younger adults, when compared with non-asthma or non-COPD age-matched control subjects, is apparent in the data; however, these patients are not yet included in vaccination recommendations. The exception is the CIQ, who state that those with asthma or COPD should be considered an at-risk population across all adult age groups and therefore should be considered for HZ vaccination [77]. HCRU and cost analyses may be required for inclusion of these patient groups in vaccination recommendations as these data are severely lacking among younger adults.

Following the recent EMA and FDA indication updates for RZV in those ≥ 18 years of age who are at increased risk of HZ, it is time to consider the increased risk populations across all age groups who may benefit from HZ vaccination.

## Data Availability

Not applicable.

## Abbreviations

ACIP: Advisory Committee on Immunization Practices
CDC: US Centers for Disease Control and Prevention
CI: Confidence interval
CIQ: Comité sur l'immunisation du Québec
COPD: Chronic obstructive pulmonary disorder
CPRD: Clinical practice research database
EMA: European Medicines Agency
FDA: U.S. Food and Drug Administration
GINA: Global Initiative for Asthma
GOLD: Global Initiative for Chronic Obstructive Lung Disease
HCP: Healthcare professional
HCRU: Healthcare resource utilization
HIV: Human immunodeficiency virus
HR: Hazard ratio
HZ: Herpes zoster
HZO: HZ ophthalmicus
IBD: Inflammatory bowel disease
ICS: Inhaled corticosteroids
IL: Interleukin
OR: Odds ratio
PHN: Post-herpetic neuralgia
RA: Rheumatoid arthritis
RR: Relative risk
RZV: Recombinant zoster vaccine
SLE: Systemic lupus erythematosus
Th: T helper
VE: Vaccine effectiveness
VZV: Varicella-zoster virus
w/: With
w/o: Without
ZVL: Zostavax^®^
